# Ponatinib‐induced ichthyosiform eruption

**DOI:** 10.1002/jha2.223

**Published:** 2021-05-14

**Authors:** Nicolas Kluger, Katriina Lappalainen, Perttu Koskenvesa

**Affiliations:** ^1^ Department of Dermatology Allergology and Venereology University of Helsinki and Helsinki University Hospital Helsinki Finland; ^2^ Department of Hematology University of Helsinki and Helsinki University Hospital Helsinki Finland

**Keywords:** chronic myeloid leukemia, dry skin, ichthyosis, ponatinib, targeted therapy

A 71‐year‐old patient with a history of chronic myeloid leukaemia (CML) diagnosed in October 2014 was referred for extensive dryness of the skin. CML was first treated with imatinib until January 2015 when it had to be stopped due to an extensive rash. Dasatinib was started at 50 mg QD. Surprisingly after 5 years of treatment with good response, the patient went into blast crisis in spring 2020. Dasatinib was replaced by ponatinib at the dose of 15 mg QD. The patient also received azacitidine cycles subcutaneously. The primary response was good but after 4 months of treatment, a *BCR–ABL1 T315I* mutation not seen at relapse was found. In November 2020, ponatinib was increased to 45 mg QD with good efficacy on CML. However, dry skin developed and worsened progressively and prompted to taper ponatinib dosage to 30 mg QD. Upon referral, the patient presented a thick grayish scaly rash with an inflammatory background that was more pronounced on the lower limbs (legs, thighs, Figures 1A and 1B) but affected also the upper limbs (Figure 1C) and shoulders, the lower abdomen and the buttocks. Head, upper trunk, palms and soles were spared. Histology of a skin biopsy from the thigh showed acanthosis and hyperkeratosis of the epidermis and some perivascular lymphocytes, but no eosinophils in the dermis (Figure 1D). Because of its efficacy on CML and the lack of alternative, ponatinib was maintained. Potent corticosteroid ointments (betamethasone 0.1% ointment) were applied along with moisturizers. Upon follow‐up, the situation has improved, and the response to ponatinib treatment is slowly improving despite the lowered dose.

**FIGURE 1:**
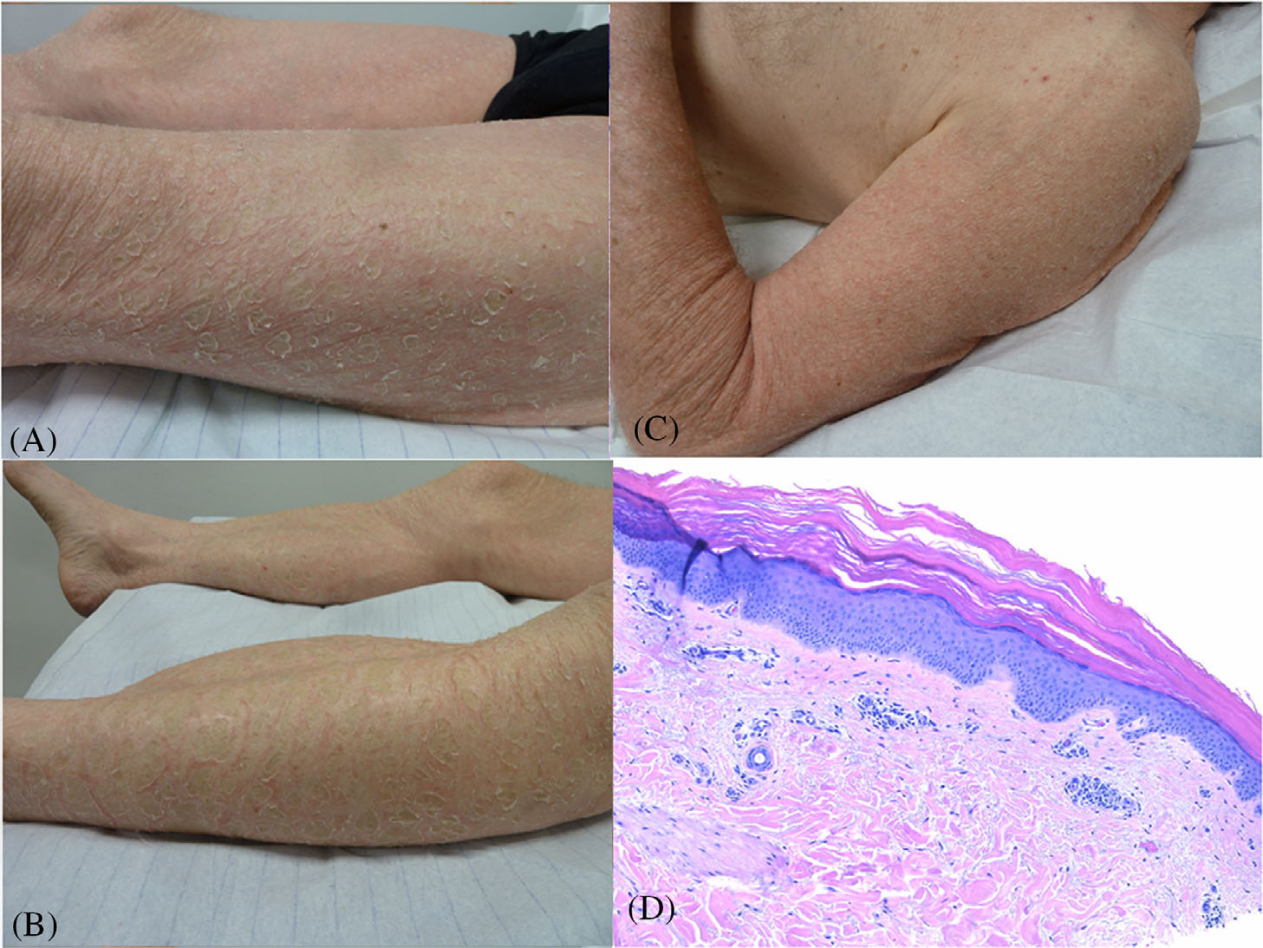
**Itchthyosiform rash under ponatinib**. Thick gray scales over an inflammatory background of the tights (A) and the legs (B). Dry inflammation of the left arm (C). Acanthosis and hyperkeratosis of the epidermis associated with a discrete lymphocytic infiltrate of the dermis (haematoxylin ‐ eosin, x 10) (D)

Ponatinib is a multikinase inhibitor that inhibits kinases other than non‐BCR‐ABL1 kinase such as FLT3, FGFR, PDGFR, KIT, RET, SRC and VEGFR. Skin rash and dry skin are extremely frequent during ponatinib phase 2 and 3 studies. Several cases of ichtyosiform rash have been described. Lesions may appear after a few weeks of treatment, sometimes even after a few days and predominate on the trunk and limbs. As in our case, an associated inflammatory component is most often reported. It is likely that the inhibition of various receptor tyrosine kinases by ponatinib, including VEGFR, FGFR and the Src family of kinases may result in the disruption of epidermal growth pathways. In conclusion, ichthysosiform rash under ponatinib can be disabling. Symptomatic measures should be put in place (emollients, keratolytics, topical retinoids, dermocorticoids if inflammation), and continuation of treatment is discussed, on a case‐by‐case basis.

## CONFLICT OF INTEREST

The authors declare that there is no conflict of interest that could be perceived as prejudicing the impartiality of the research reported.

## PATIENT CONSENT STATEMENT

The patient gave his informed consent for the use of the pictures and the publication of this case report.

